# Description of a Naphthoquinonic Crystal Produced by the Fungus *Scytalidium cuboideum*

**DOI:** 10.3390/molecules23081905

**Published:** 2018-07-31

**Authors:** Sarath M. Vega Gutierrez, Kenya K. Hazell, John Simonsen, Seri C. Robinson

**Affiliations:** Wood Science & Engineering, Oregon State University, Corvallis, OR 97331, USA; hazellk@oregonstate.edu (K.K.H.); John.simonsen@oregonstate.edu (J.S.); seri.robinson@oregonstate.edu (S.C.R.)

**Keywords:** organic crystal, naphthoquinone, *Scytalidium cuboideum*, XRD, TEM, NMR

## Abstract

Intarsia was an art form popular between the 15th–18th centuries that used wood pigmented by spalting fungi to create detailed landscapes, portraits, and other imagery. These fungi are still used today in art but are also finding relevance in material science as elements of solar cells, textile dyes, and paint colorants. Here we show that the spalting fungus *Scytalidium cuboideum* (Sacc. and Ellis) Sigler and Kang produces a red/pink pigment that forms two distinct colors of crystals (red and orange)—a very rare occurrence. In addition, a second structure of the crystal is proved through nuclear magnetic resonance (NMR). This is only the second instance of a stable, naphthoquinone crystal produced by a fungus. Its discovery is particularly valuable for solar cell development, as crystalline materials have a higher electrical conductivity. Other fungi in this order have shown strong potential as thin films for solar cells.

## 1. Introduction

In the 1400s, intarsia art (a type of marquetry) spread across Europe. This technique used colored wood inlays to create complex paneling, furniture, and other decorative objects for the elites of that era [1,2]. The natural and synthetic color palette was limited, and woodworkers often turned to wood already colored by fungi (spalted wood) to add rich blue-greens, slate blues, reds, and black lines to their work. Spalted wood was used routinely in marquetry work in Europe until the arrival of the industrial revolution, when the synthesis and cost-effectiveness of chemical dyes overtook natural dyes. Interestingly, the spalted pigments used in old marquetry woods are still vibrant and show no fading, while the synthetic pigments and other natural colorants used faded over time [3,4]. Spalted wood remains popular today in wood art, particularly woodturning [5], due to its long-lasting color stability.

Spalting fungi are also of increasing interest to scientists for the unique secondary metabolites (pigments) that are produced. These pigments have shown promise as films in solar cells [6,7,8], dye additives in paints and finishes [9], and as UV-stable, water-resistant textile dyes [10,11].

One of these spalting fungi, *Scytalidium cuboideum* (Sacc. and Ellis) Singler and Kang, produces a red pigment called Draconin Red [10]. It was first identified chemically by Golinski et al. in 1995 [12], and is capable of colonizing both wood and bamboo [13], in addition to being used as a textile dye [10,11] and paint colorant [14]. Recent research on this pigment has shown crystal-like structures [15]; however these structures have not been confirmed nor determined to be real crystals.

General crystals produced by fungi are not uncommon. There are several studies on calcium oxalate crystals produced by white rot fungi [16], and several fungal pathogens of woody materials produce pigments that crystallize, such as the shiraiachromes [17]. However, natural naphthoquinone crystals are very rare. They occur in only a few bacterial species such as *Actinoplanes* sp. [18]; can be induced in some *Fusarium* species in fungi [19]; and others. Specifically for spalting fungi, xylindein naphthoquinone crystals can be synthesized from derivatives of this pigment, but the addition of chemicals, such as phenols, are required [20,21]. In contrast, the assumed crystals of *Scytalidium cuboideum* are produced directly by the fungus and can be obtained by a simple cold acetone extraction. No further processing or intervention is required.

The existence of naturally occurring crystal pigment has potential commercial applications but is specifically of interest in the solar energy field, as crystals often have a higher potential for electrical conductivity. The Draconin Red pigment has never been investigated for solar cell use, unlike the pigment produced from *Chlorociboria* species (xylindein), which makes excellent thin films for solar cells but does not form crystals [6]. Although unrelated, Draconin Red shares many properties with xylindein, such as light stability, poor solubility, and excellent bioadhesion [10].

Herein, we detail the methodology and identification of a naturally-occurring naphthoquinone crystal, produced from the spalting fungus *S. cuboideum*. This crystal and its unique structure should shed new light into the light stability of Draconin Red, and allow scientists to further explore its potential applications in not only the textile and paint industries, but also as a potential medium for green energy production.

## 2. Materials and Methods

### 2.1. Transmission Electron Microscopy

Kikuchi patterns are a specific pattern for crystal materials and can be compared to a fingerprint for crystalline materials [22]. With transmission electron microscopy, TEM, it is possible to perform an electron diffraction analysis in search of Kikuchi patterns [23]. To perform this experiment, an FEI Titan 80–200 (Thermo Scientific, Hillsboro, OR, USA) was used. Extracted pigment from *Scytalidium cuboideum* (dissolved in dichloromethane (DCM (Merck, Kenilworth, NJ, USA))) was obtained by following the protocol of Robinson [24]. Three drops of this pigment were applied into a Silicon-Nitride TEM grid (Ted Pella Inc., Redding, CA, USA) that was previously polished with a PELCO easiGlow™ plasma cleaner (Ted Pella Inc., Redding, CA, USA). After allowing for the evaporation of the DCM, the grid was inserted into the sample holder and placed into the TEM, at which point the diffraction analysis was performed.

### 2.2. Crystallization

The crystals of pigment were obtained by precipitation—achieved by applying liquid nitrogen to the pigments suspended in a solvent with a low melting point. The solvent chosen for this experiment was Millipore Sigma acetone HPLC (melting point −95.4 °C) (VWR, Radnor, PA, USA). Liquid nitrogen (200 mL) (OSU Chemistry Stores, Corvallis, OR, USA) was applied to the pigment, suspended in 100 mL of acetone, to achieve a cold state (−60 °C) that allowed the pigment to crystallize. After the crystals were formed in the cold solvent, they were filtered with the use of VMR filter paper 415 (VWR, Radnor, PA, USA). The crystals were then air-dried and placed in a glass vial.

### 2.3. Light Microscopy

Light microscopy was used to observe the morphology and color of the crystals obtained. For this, 0.5 g of the dry crystals were dispersed in 15 mL of distilled water. Two drops of the solution were applied on a VWR plain glass slide and covered with a VWR glass cover slip No. 1 (VWR, Radnor, PA, USA). Images of the crystals were obtained with a Nikon Eclipse Ni-U equipped with a Nikon DS-Ri2 camera and the Nikon NIS-Elements Fr software (Nikon Instruments Inc., Melville, NY, USA).

### 2.4. Scanning Electron Microscopy (SEM)

A silicon chip specimen support of 5 × 7 mm (Ted Pella, Inc., Redding, CA, USA) was used as a base to apply resolubilized crystals in Millipore Sigma acetone HPLC (VWR, Radnor, PA, USA). Five drops of the solution were applied onto the silicon support. Each drop was left to be fully evaporated before the next application. After the five drops were completed, the silicon support was fixed to a Ted Pella, Inc. (Ted Pella, Inc., Redding, CA, USA) aluminum stud of one centimeter in diameter for SEM. Ted Pella, Inc. double-coated carbon conductive tape was used to fix the samples to the aluminum studs.

Then, a sputter coating of 30–45 nm (deposited after an exposure of 35 s) gold-palladium was applied with a Cressington Sputter Coater 108 Auto (Cressington Scientific Instruments, Inc., Watford, UK) to give the samples an enhanced optical contrast. Then, the sample was placed in an FEI QUANTA 600F environmental SEM (FEI Co., Hillsboro, OR, USA). The samples were viewed at an electron spot size of 2.5 and a high voltage (HV) of 5 kV.

### 2.5. X-ray Crystallography

Diffraction intensities for single *S. cuboideum* crystals (orange and red) were collected at 173 K on a Bruker Apex2 CCD diffractometer (Bruker, Portland, OR, USA) using an Incoatec Cu *IµS* source, Cu Kα radiation, 1.54178 Å. The space group was determined based on systematic absences. Absorption correction was applied by SADABS [25]. Structures were solved by direct methods and Fourier techniques and refined on *F*^2^ using full matrix least-squares procedures. All non-H atoms were refined with anisotropic thermal parameters. H atoms were found on the residual density map and refined with isotropic thermal parameters. All calculations were performed by the Bruker SHELXL-2014/7 packages [26] (Bruker, Portland, OR, USA).

### 2.6. Nuclear Magnetic Resonance (NMR)

Each of the data sets were collected on a Bruker AVIII 700 (Bruker, Portland, OR, USA), running Topspin 3.5PL7. The probe head employed was a DCH cryo-probe, Dual Nucleus C13 and H1 with H2 lock and Z gradient. Several samples were submitted, and the data presented drew from multiple samples. All of the samples were dissolved in CDCl_3_ from CIL. The H1 and C13 chemical shift references were 7.24 from H1 and 77.23 for C13.

The geometry optimized structures for Isomer A and B were produced with Spartan 10, and the tautomeric structure was generated using Chemdraw Ultra V 12.0.2.1076 (PerkinElmer, Richmond, CA, USA).

## 3. Results

### 3.1. TEM

**Figure 1:**
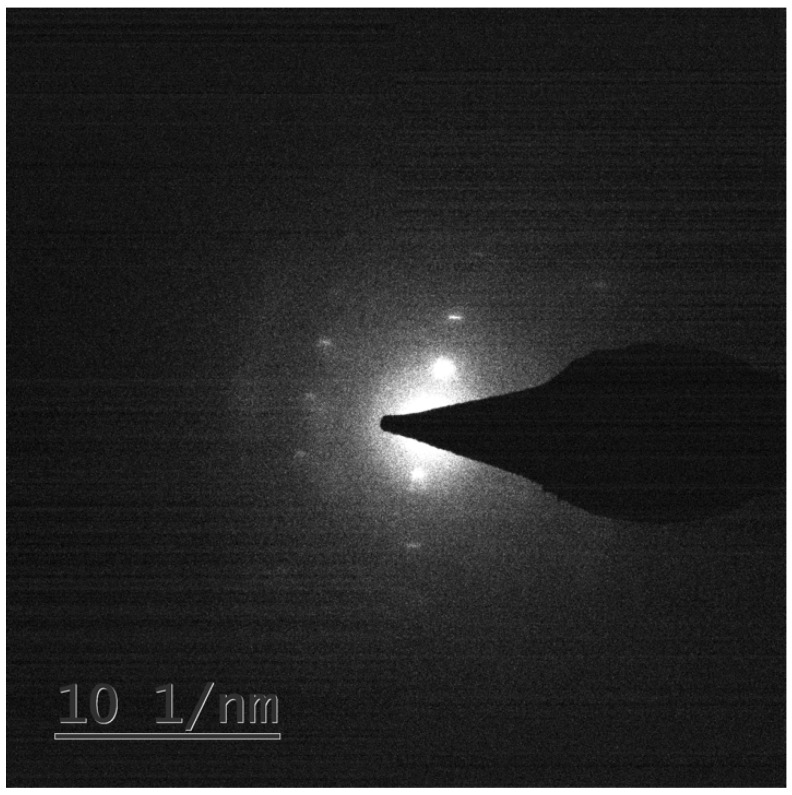
Kikuchi pattern obtained from the extracted pigment from *S. cuboideum* with an FEI Titan 80–200.

A Kikuchi pattern was established, see Figure 1, confirming the crystal nature of the pigment of *S. cuboideum*. A detailed search of the obtained Kikuchi pattern and the chemical composition of the pigment was performed in several diffraction databases such as the Crystallography Open Database, the International Center for Diffraction Data, Cambridge Structural Database, and SciFinder Scholar with no success. This indicates that the crystal produced by the pigment of *S. cuboideum* has a high probability of being a novel organic crystal. The tentative name given to this crystal was “Dramada”, and an X-ray diffraction (XRD) study was performed to confirm its discovery.

### 3.2. Light Microscopy and SEM

**Figure 2:**
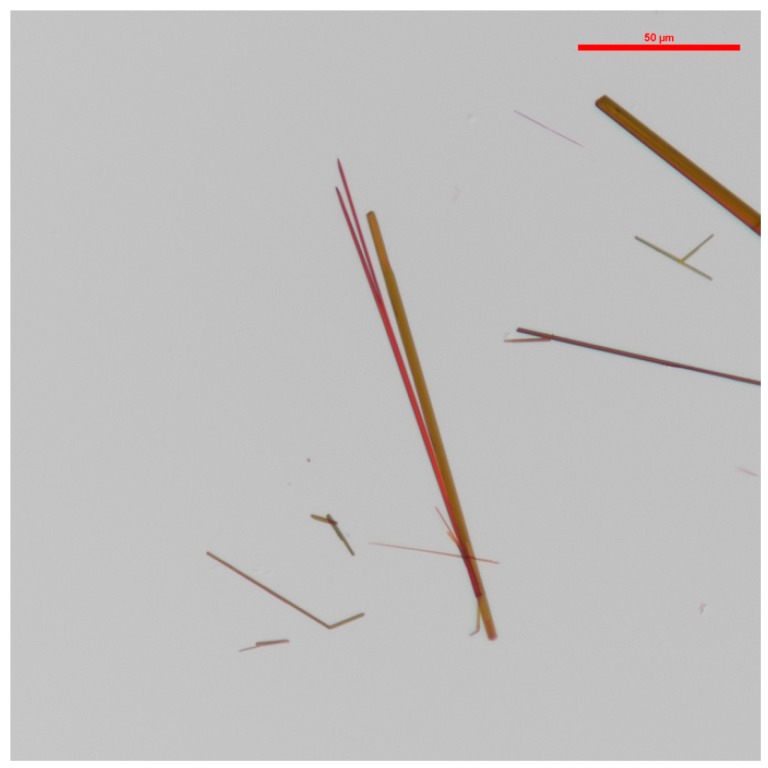
Red and orange coloration of the crystals produced by the pigment of *S. cuboideum*. Image obtained with a Nikon Eclipse Ni-U at a magnification of 20×.

Light microscopy shows the crystals have a ‘’needle-like‘’ longitudinal appearance. They occurred in both red and orange hues, as shown in Figure 2.

**Figure 3:**
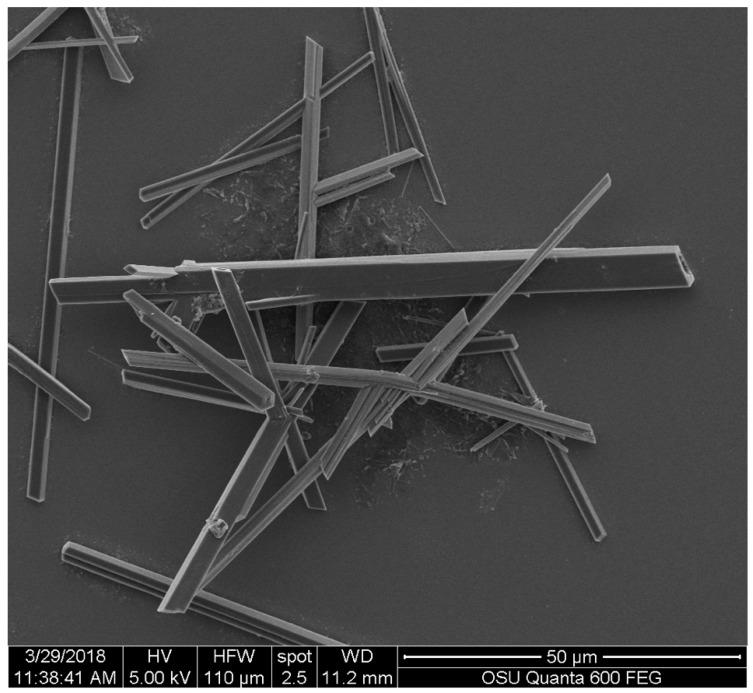
Scanning electron microscopy (SEM) image of the crystals produced by the pigment of *S. cuboideum* detailing their angular nature. Image obtained with an FEI QUANTA 600F environmental SEM.

The SEM image allowed a more detailed observation of the appearance of the crystals. In Figure 3, it is possible to observe the rectangular cross-section of the crystals and the angular detail along the ‘’needle-like‘’ structures.

### 3.3. Crystallographic Data for Dramada

**Figure 4:**
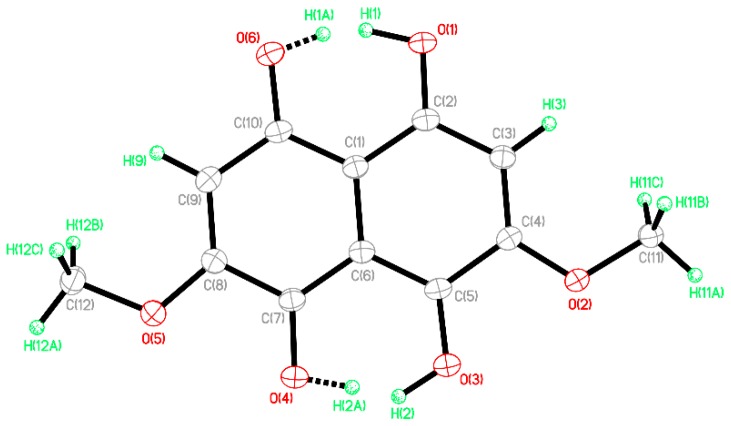
Chemical structure of *Dramada* obtained with X-ray diffraction (XRD).

The data obtained with XRD gave the following values for the crystal: C_12_H_10_O_6_, M = 250.20, 0.14 × 0.07 × 0.05 mm, T = 173(2) K, Monoclinic, space group *P*2_1_/*c*, *a* = 3.8826(3) Å, *b* = 30.971(3) Å, *c* = 9.1803(8) Å, *β* = 100.435(6)°, *V* = 1085.67(17) Å^3^, *Z* = 4, *D*c = 1.531 Mg/m^3^, μ(Cu) = 1.072 mm^−1^, F(000) = 520, 2θ_max_ = 133.18°, 7232 reflections, 1909 independent reflections (R_int_ = 0.0371), R1 = 0.0362, wR2 = 0.0934 and GOF = 1.041 for 1909 reflections (211 parameters) with *I* > 2*σ*(*I*), R1 = 0.0435, wR2 = 0.0989 and GOF = 1.041 for all reflections, max/min residual electron density +0.147/−0.230 eÅ^−3^. The proposed structure obtained with XRD is presented in Figure 4.

From the crystallographic results, it was found that -OH groups in the crystal were disordered in a 1:1 ratio over two positions, corresponding to two possible orientations of the molecules in the crystal structure. This structure was identical for the red and orange crystals. Due to the particular nature of the molecule found with XRD, further analysis was performed with NMR, obtaining the following results.

### 3.4. Nuclear Magnetic Resonance (NMR)

**Figure 5:**
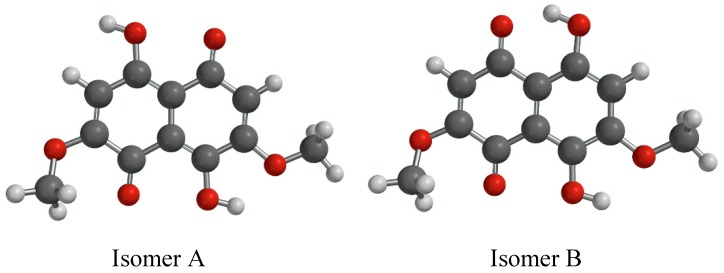
Nuclear Magnetic Resonance (NMR) proposed structures.

The proposed structure was presumed to be one of the two regional isomers indicated below in Figure 5 as Isomers A and B.

**Figure 6:**
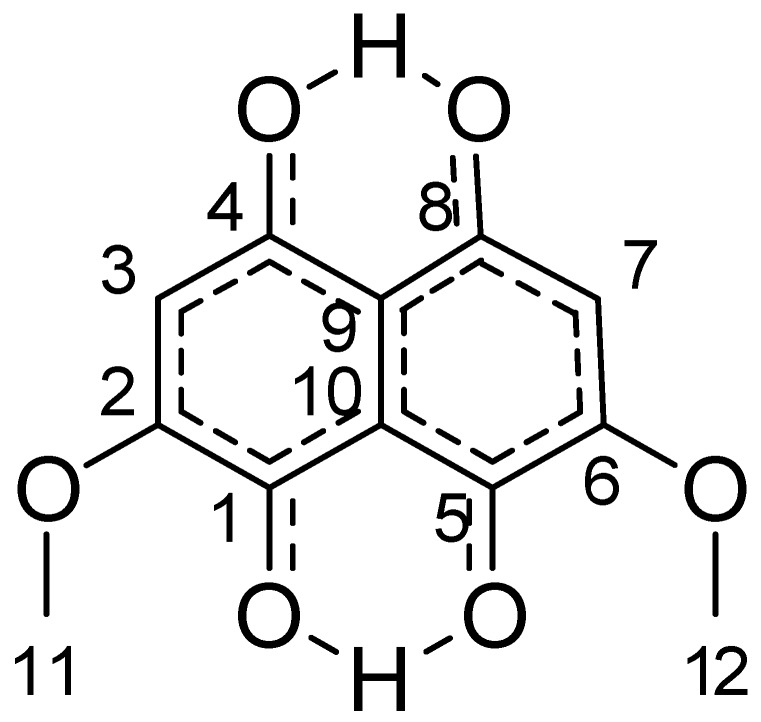
Tautomer structure of 3,6 di-methoxy (di-hydroxyl-napthoquinone).

The 1D H1 spectrum below showed only four relevant signals. It showed two inequivalent OH type protons at 13.14 ppm and 12.71 ppm, two equivalent CH protons at 6.38 ppm, and six equivalent protons as 3.93 ppm as two O methyl groups (O-CH_3_). Comparison of the proton data with isomers A and B removed both of those from consideration as the CH and O-CH moieties were inequivalent. This was the same for the other regional isomers that could not be modeled. These remain consistent with the molecular formula from the complimentary high-resolution mass spectrometry (MS) that was performed. It is proposed that the structure of the compound exists as a tautomer, depicted in Figure 6. This structure was similar to the one obtained with the use of XRD.

The assignments that follow were from H1, Supplementary Materials Figure S1, and C13, Supplementary Materials Figure S2, identification spectra; along with HSQC (Hetero-Nuclear Single Quantum Coherence), Supplementary Materials Figure S3; HMBC (Hetero-Nuclear Multiple Bond Coherence), Supplementary Materials Figure S4 a 1,1-ADEQUATE (1,1-AD) all H1/C13, Supplementary Materials Figure S5; and the 2D H1 NOESY, Supplementary Materials Figure S6.

From the HSQC correlations at 3.93 ppm H1 and 56.92 ppm C13 with an assigned C11 and C12, the correlations at 6.38 ppm and 109.5 ppm were assigned C3 and C7. Using the HMBC H1 correlations from the proton (13.14) proximal to C4,8 was assigned C9, C4,8 and verified C3,7 at 104.39 ppm, 173.18 ppm, and 109.5 ppm, respectively. The proton (12.72) proximal to C1,5 supplied the next set of HMBC contacts to assign the balance of the carbons. Assigned are C10 at 112.1 ppm, C1,5 at 167.34 ppm and C2,6 at 159.0 ppm. The aromatic CH proton at 6.39 ppm had strong J3 (3 bonds) correlations at 109.5 ppm and 167.34 ppm, corresponding to C3,7 and C1,5, respectively. The CH_3_ methyl resonance at 3.93 ppm showed a single strong J3 correlation to C2,6, confirming that assignment.

A 1,1-ADEQUATE experiment was also performed. This experiment generally only shows J2 H1-C13 correlations. This test was used in a complementary manner with the HMBC that determined J3 H1-C13 couplings. The only expected correlation would be from the 6.36 ppm aromatic proton as all other proton types would have 0.16 ppm in the molecular orbital of interest. The 1,1-AD shows three correlations, an HSQC breakthrough at 109.5 ppm, and then two J2 signals at C2,6 at 159.0 ppm and C4,8 at 173.18 ppm, confirming the J2 of the atom types.

NOESY data was initially considered as being essential to distinguish between the original pair of regio-isomers; therefore, it was collected. There were NOE correlations between the pair of OH type protons at 13.14 ppm and 12.71 ppm to the O methyl CH_3_ at 3.93 ppm. The inter-nuclear distances between those atoms types were approx. 5.4 Å and 6.4 Å respectively. The NMR spectra are attached in their entirety in the supplementary materials.

## 4. Discussion

As the crystal produced by *S. cuboideum* has a naphthoquinone nature, it is likely one of the few naphthoquinone crystals produced by a fungus. The other microbial-produced naphthoquinones come from some species in the *Fusarium* genus [19], as well as the bacterial genus *Actinoplanes*, both of which require phenols [20] in addition to chlorinated chemicals [19] to synthesize. In contrast, the *S. cuboideum* crystals were produced with an acetone extraction and by precipitation with liquid nitrogen. This method of synthesis can be applied to a larger scale and without the requirement of further compounds that can be harmful to the operators. Also, the ability of this fungus to produce pigments that can be easily crystallized opens a new door for further applications of an easily produced renewable pigment.

Pharmacological applications may also be possible, noting the nature of this pigment. The industry already uses the naphthoquinone crystals obtained from the bacteria *Actinoplanes* (despite their difficulty in production) as an antibiotic [18,27,28,29]. To determine the potential use of the novel crystals of *S. cuboideum*, further toxicological testing would be required.

The crystals also presented two distinct colors (red and orange), distinguished via light microscopy. This difference in color may indicate that the fungus produces two different types of crystalline compounds, or that the molecule described by [12] has an isomer that reflects light differently, as also occurs with xylindein (from *Chlorociboria* species, amusingly, also a very well-known spalting fungus) [20]. This color difference could also be related to the -OH groups that can cause light to be diffracted in different wavelengths, resulting in the optical visualization of the two colors. The presence of two different crystal colorations could lead to the development of a broader color palette for the fungal crystals, resulting in a richer variety of dyes.

### 4.1. XRD and NMR

Due to the positioning of the -OH groups present in the XRD, the analysis proved inconclusive. Therefore, NMR was required to obtain a final structure.

NMR showed that the spectra were consistent with the tautomeric structure presented in Figure 5 and also with the XRD molecule presented in Figure 3. There were no extraneous or uncorrelated signals. While NMR results are always presented as being consistent with a structure and not an isomorphic map between structure and data set, it is clear that no variation of region isomerization was consistent with even the 1D data. Instead, the collective data was consistent with the identified structure. Additionally, it is not generally expected that NOE correlations will have an inter-nuclear distance greater than 4 Å. This is explained as being due to a proton exchange process as the relative intensities were consistent with inter-atomic distance. This structure was complicated by its proton deficient nature, exchangeable protons, and the degree of symmetry. Several attempts were made to methylate the exchangeable OH protons in the belief that this would yield a structure more amenable to NMR analysis. Those attempts were met with varying degrees of success. Eventfully it was realized that likely as a product of the enzymatic synthesis of the secondary metabolism of *S. cuboideum* in an aqueous medium, the compound only ever existed as a tautomer, at least on the time scale required for NMR observation. If the parent structure requires unequivocal determination at a later point, a combination of QM transition state calculation and observation via solid-state NMR of the crystal material would be advisable.

### 4.2. Application

The red pigment from *S. cuboideum* has been successfully utilized across a number of commercial products, including as a textile dye [10,11,30,31], a paint colorant [14,32], and a wood dye [13,24,33]. Unfortunately, the pigment has always been suspended in DCM (a problematic and toxic solvent), as when the pigment is non-solubilized it binds to its substrate and is unusable. Having the pigment in a crystal form produces a sort of clumpy powder that is bound mostly to itself. This makes the pigment easy to add into oils, paints, and other solvents as desired.

Interestingly, it is the crystalline nature of this pigment that appears to make it such a stable colorant. Crystals are highly stable, due to strong intermolecular bonding. The crystalline structure is also likely to result in reduced reactivity of the compound, making it more stable than an amorphous form. For example, previous work by Vega Gutierrez [15] found that the red pigment wrapped around polyester fibers, which decreased the color loss after washing, crocking, and bleaching [10]. Further research on tested samples showed that the spooling of the pigment was actually the formation of long, winding crystals. Additionally, the crystal phase resulted in a stable chemical composition with higher purity compared to the DCM extraction from amended wood malt agar plates [34]. This crystal phase opens the door for further research into variables such as pressure and temperature, which could be used to modify the properties and behavior of the crystal and make it useful for additional applications [35].

The discovery of this crystal would have been improbable if not for the regained interest in the ancient art of intarsia, and the fungi responsible for the long-lasting wood colors [1,4], as well as the continued movement towards natural, stable colorants for wood, textiles [10,13,24], biomarkers, and solar cells [6]. The discovery of the *S. cuboideum* crystal sheds new light on the color stability of an ancient art form and offers a look at how this naturally occurring, renewable pigment can contribute to modern colorants and their industry potential.

## 5. Conclusions

The regained interest in the ancient art of intarsia has shed light on the potential of spalting fungal pigments, not as simple metabolites, but as materials. Within this research, it was determined that the pigment extracted from *S. cuboideum* produces a novel, organic crystal with a naphthoquinone composition in two different colors. These crystals can be obtained with simple extraction methods and represent new and wider possibilities for dye-based industries which are seeking highly stable, long-lasting, natural colorants that can compete within the synthetic market, as well as within the solar energy industry.

## Supplementary Materials

The following are available online: Figure S1–S6.
