# Supplementary material for: Description of a Naphthoquinonic Crystal Produced by the Fungus Scytalidium cuboideum
Source: Molecules. 2018 Jul 31;23(8):1905. doi: 10.3390/molecules23081905 (PMC6222619; doi:10.3390/molecules23081905)
Supplement: Supplementary file 1 [file molecules-23-01905-s001.pdf]

Description of a Naphthoquinonic Crystal Produced by the fungus *Scytalidium cuboideum*

Sarath M. Vega Gutierrez*, Kenya K. Hazell, John Simonsen and Seri C. Robinson

Wood Science & Engineering Oregon State University; hazellk@oregonstate.edu (K.K.H.); John.simonsen@oregonstate.edu (J.S.); seri.robinson@oregonstate.edu (S.C.R.)

* Correspondence: Sarath.vega@oregonstate.edu; Tel.: +xx-xxx-xxx-xxxx

Received: date; Accepted: date; Published: date

Supplementary Materials:

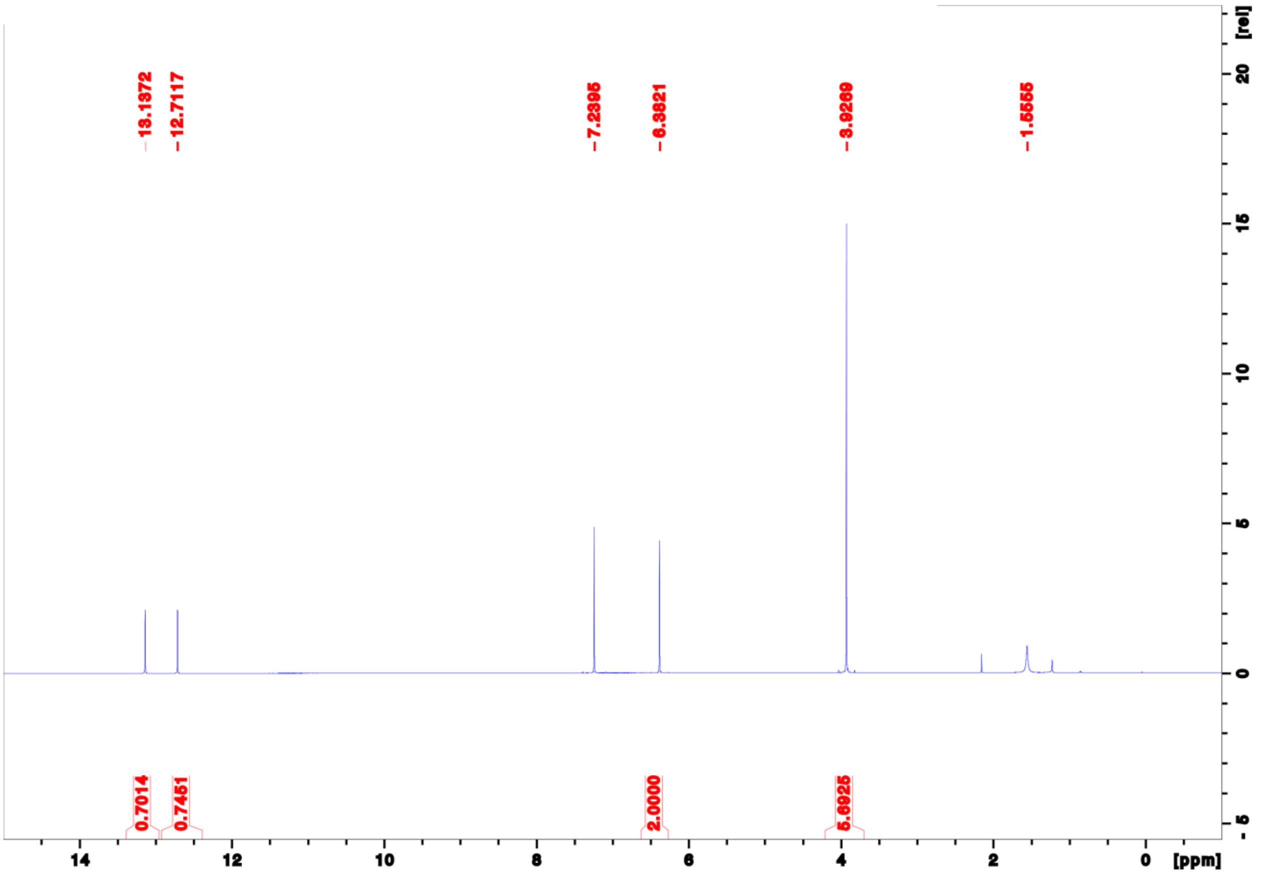

S1. ^{1}H ($CDCl_3$) spectrum of the new compound 'Dramada'.

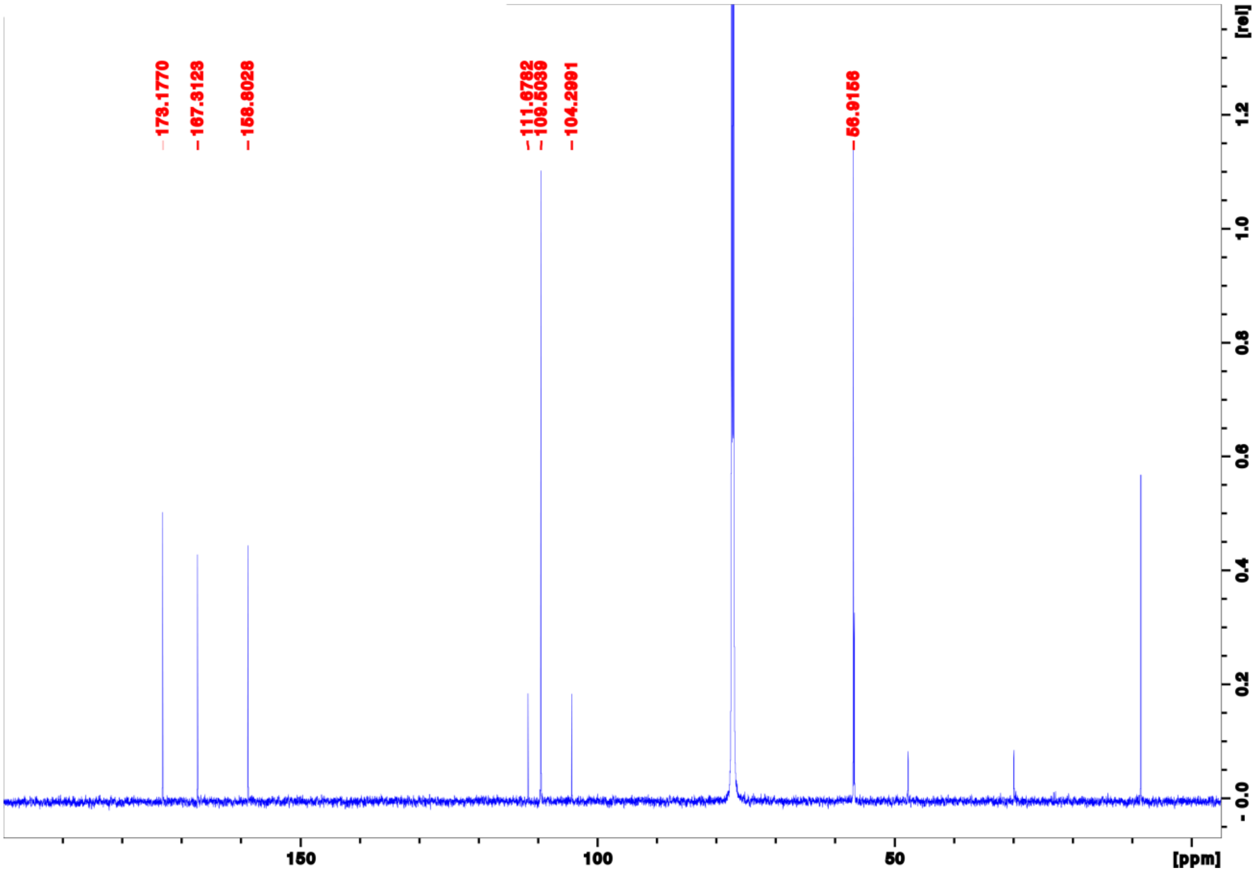

S2. ^{13}C ($CDCl_3$) spectrum of the new compound ‘Dramada’.

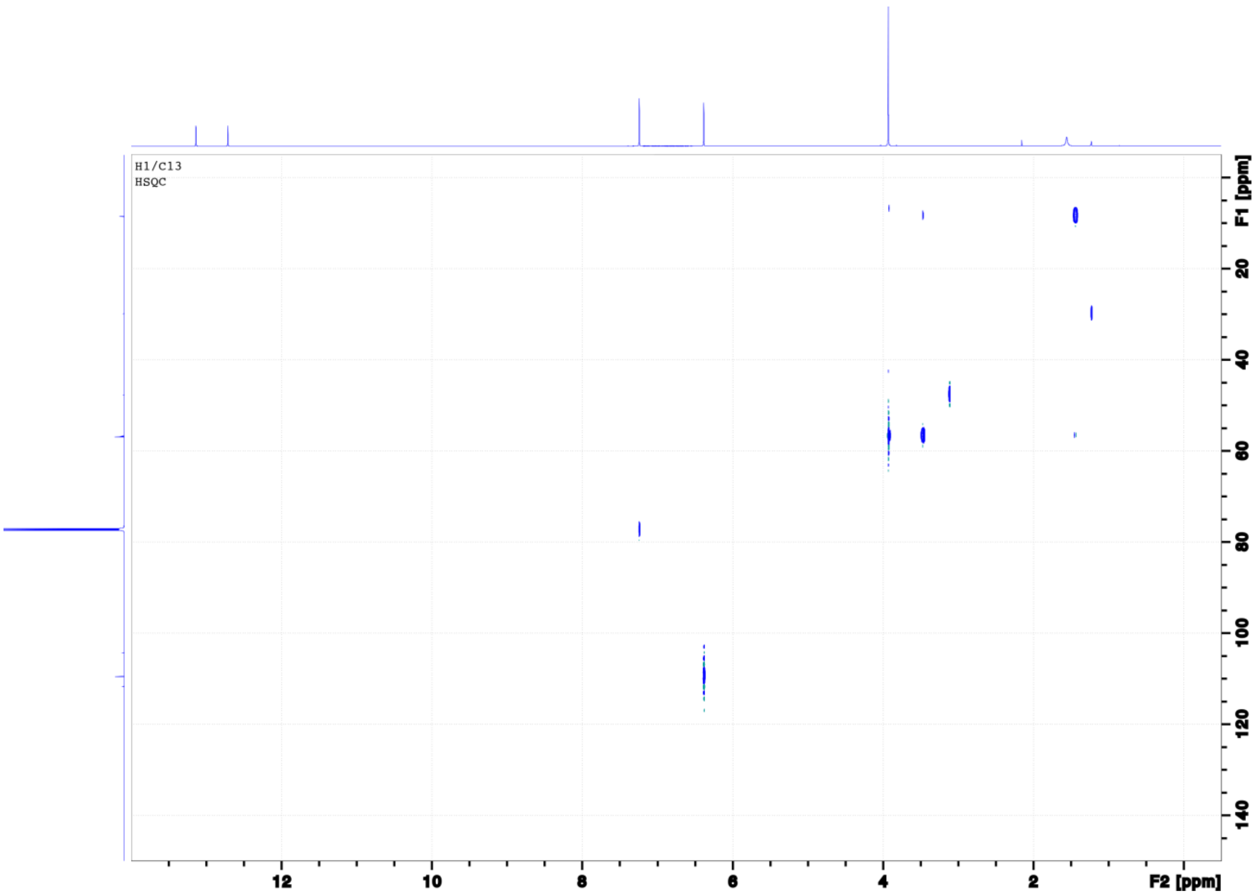

S3. Hetero-nuclear single quantum coherence (HSQC) ($CDCl_3$) spectrum of the new compound 'Dramada'.

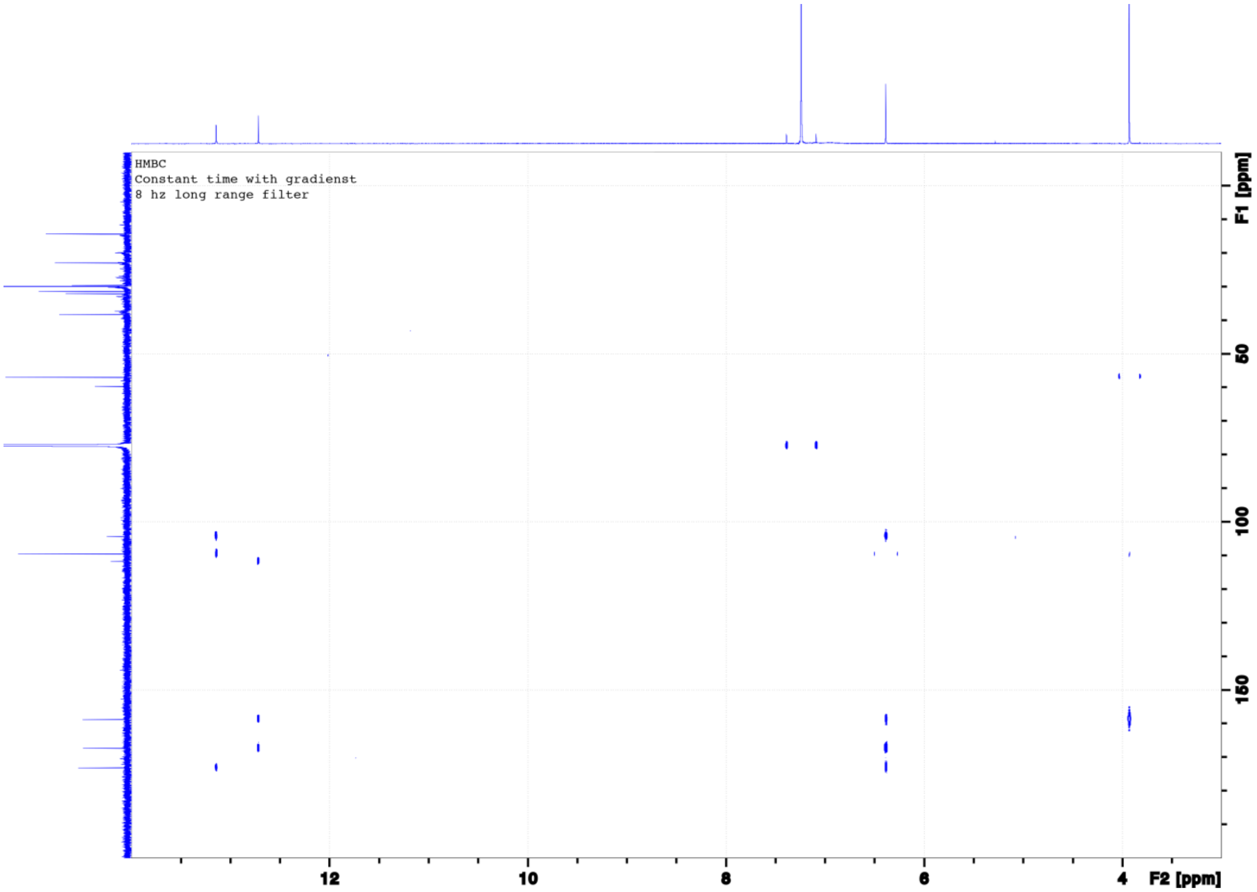

S4. Hetero-nuclear multiple bond coherence (HMBC) ($CDCl_3$) spectrum of the new compound 'Dramada'.

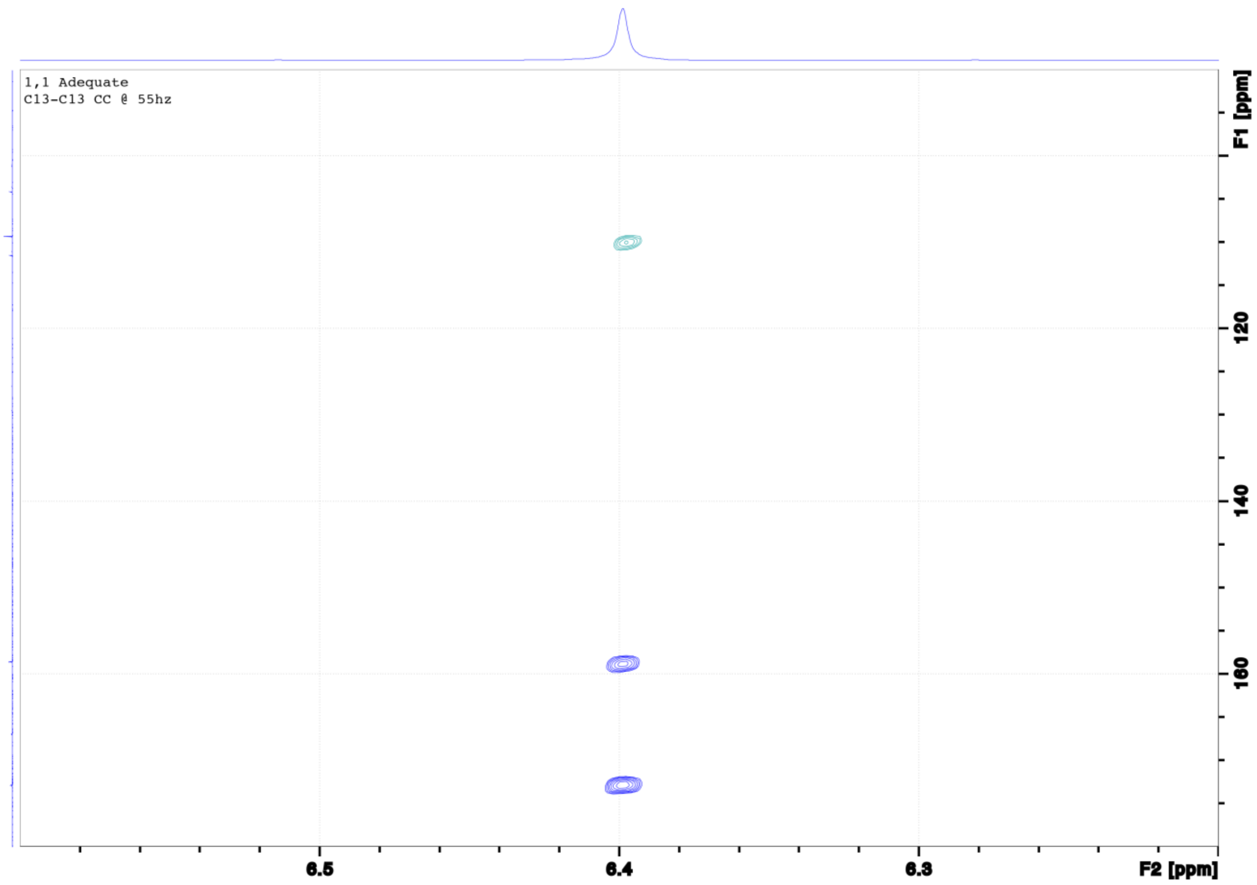

S5. ^{1,1} Adequate ($CDCl_3$) spectrum of the new compound ‘Dramada’.

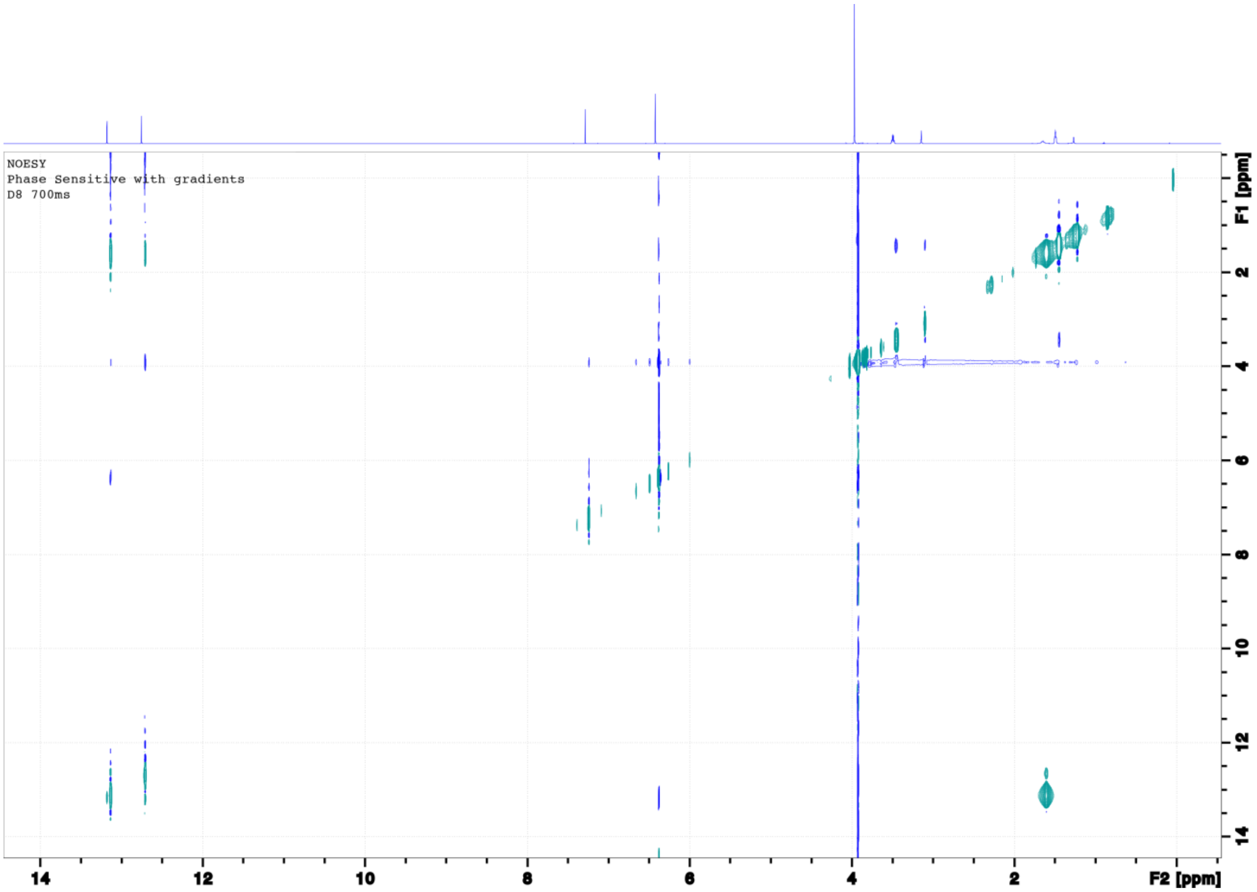

S6. NOESY ($CDCl_3$) spectrum of the new compound ‘Dramada’.
